# Two Faces of Fermented Foods—The Benefits and Threats of Its Consumption

**DOI:** 10.3389/fmicb.2022.845166

**Published:** 2022-03-07

**Authors:** Krzysztof Skowron, Anna Budzyńska, Katarzyna Grudlewska-Buda, Natalia Wiktorczyk-Kapischke, Małgorzata Andrzejewska, Ewa Wałecka-Zacharska, Eugenia Gospodarek-Komkowska

**Affiliations:** ^1^Department of Microbiology, Ludwik Rydygier Collegium Medicum in Bydgoszcz, Nicolaus Copernicus University in Toruń, Bydgoszcz, Poland; ^2^Department of Hygiene, Epidemiology, Ergonomy and Postgraduate Education, Ludwik Rydygier Collegium Medicum in Bydgoszcz, Nicolaus Copernicus University in Toruń, Bydgoszcz, Poland; ^3^Department of Food Hygiene and Consumer Health, Wrocław University of Environmental and Life Sciences, Wrocław, Poland

**Keywords:** contaminated fermented products, fermented foods, food safety, foodborne, outbreaks

## Abstract

In underdeveloped and developing countries, due to poverty, fermentation is one of the most widely used preservation methods. It not only allows extending the shelf life of food, but also brings other benefits, including inhibiting the growth of pathogenic microorganisms, improving the organoleptic properties and product digestibility, and can be a valuable source of functional microorganisms. Today, there is a great interest in functional strains, which, in addition to typical probiotic strains, can participate in the treatment of numerous diseases, disorders of the digestive system, but also mental diseases, or stimulate our immune system. Hence, fermented foods and beverages are not only a part of the traditional diet, e.g., in Africa but also play a role in the nutrition of people around the world. The fermentation process for some products occurs spontaneously, without the use of well-defined starter cultures, under poorly controlled or uncontrolled conditions. Therefore, while this affordable technology has many advantages, it can also pose a potential health risk. The use of poor-quality ingredients, inadequate hygiene conditions in the manufacturing processes, the lack of standards for safety and hygiene controls lead to the failure food safety systems implementation, especially in low- and middle-income countries or for small-scale products (at household level, in villages and scale cottage industries). This can result in the presence of pathogenic microorganisms or their toxins in the food contributing to cases of illness or even outbreaks. Also, improper processing and storage, as by well as the conditions of sale affect the food safety. Foodborne diseases through the consumption of traditional fermented foods are not reported frequently, but this may be related, among other things, to a low percentage of people entering healthcare care or weaknesses in foodborne disease surveillance systems. In many parts of the world, especially in Africa and Asia, pathogens such as enterotoxigenic and enterohemorrhagic *Escherichia coli, Shigella* spp., *Salmonella* spp., enterotoxigenic *Staphylococcus aureus, Listeria monocytogenes*, and *Bacillus cereus* have been detected in fermented foods. Therefore, this review, in addition to the positive aspects, presents the potential risk associated with the consumption of this type of products.

## Introduction

Fermentation is one of the oldest processes that allows the preservation of food stability with the participation of microorganisms. The term itself comes from the Latin word fervere, which means “to cook.” The action of microorganisms is based on the breakdown of complex compounds (carbohydrates and other macromolecules) into simple ones, which is accompanied by the formation of various types of beneficial catabolites, such as B vitamins, minerals, or Omega-3 fatty acids (Sivamaruthi et al., 2018). In most fermented products, lactic acid bacteria (LAB) play a major role in production. Also, several dozen types of bacteria, yeast, and filamentous fungi participate in the food fermentation (Laranjo et al., 2017; Rezac et al., 2018; Dimidi et al., 2019; van Reckem et al., 2019; Vilela et al., 2020; Zang et al., 2020; García-Díez and Saraiva, 2021; Xu et al., 2021).

A variety of single or mixed raw materials of plant origin (including cereals), meat and fish, and dairy products can be fermented. Such food can be eaten as a main course, drink, or snack.

Fermented foods can include processed foods on a small scale (household, craft industry) and large scale (industrially processed foods). A relevant role in traditionally fermented food play available plant or animal raw materials but also the customs, culture, and religion of indigenous peoples. Techniques of the fermentation process in some geographic areas are passed only orally, from generation to generation, and therefore are known to communities living close to each other (Anyogu et al., 2021).

Since ancient times, fermented foods have been produced by a process of natural (wild, spontaneous) fermentation, carried out by indigenous microorganisms naturally present in the raw material or processing environment (Campbell-Platt, 1987). The dominance of fermenting microorganisms, their metabolites and the changing pH of the raw material inhibit the growth of pathogenic microorganisms. Natural fermentation occurs also when a component containing a large number of microorganisms that initiate the fermentation process is added to the raw material. In both cases, the microorganisms involved in fermentation and the microclimate impact a product quality. The backsloping method, involving the use of a previously fermented product to inoculate a new batch, has also been used. This approach increases the chances of the desired microorganisms domination and competition with microorganisms that responsible for the product spoilage or disease. These traditional fermentation methods are still used today, primarily in home-based, local food production, or small-scale production. However, in the twentieth century, the development of microbiology, including food microbiology, has led to starter cultures introduction, which initiate the fermentation process and at the same time ensure greater product standardization. Such method results in products with constant organoleptic properties. Fermentation with well-defined cultures has found application, especially in the case of products obtained on an industrial scale. The process conducted under controlled conditions, it allows increasing the pace of the process and its throughput. The predominance of native microbiota allows limiting the growth of undesirable strains or species of microorganisms, as well as to reducing the toxic compounds they produce, ensuring the food safety. In developed countries, fermentation with the use of starter cultures also aims to achieve new health goals (Hesseltine and Wang, 1980; Holzapfel, 1997; Tamang et al., 2016, 2020; Voidarou et al., 2020; Mannaa et al., 2021). Research aimed at improving the starter cultures properties, carried out using the innovative CRISPR/Cas9 technology (Clustered Regularly Interspaced Short Palindromic Repeats/CRISPR-associated protein 9) which allows modification of the genome of any microorganism, may play an essential role here (Wu et al., 2020; Vilela, 2021). On the other hand, due to the current trend toward organic and biodynamic production, and the “flat” taste of products made with the participation of bacterial and fungal starter cultures, the strategy of traditional, spontaneous fermentation, and artisanal returns. This, however, increases the risk of the dangerous microorganisms presence in food (Capozzi et al., 2017).

## Benefits

**FIGURE 1:**
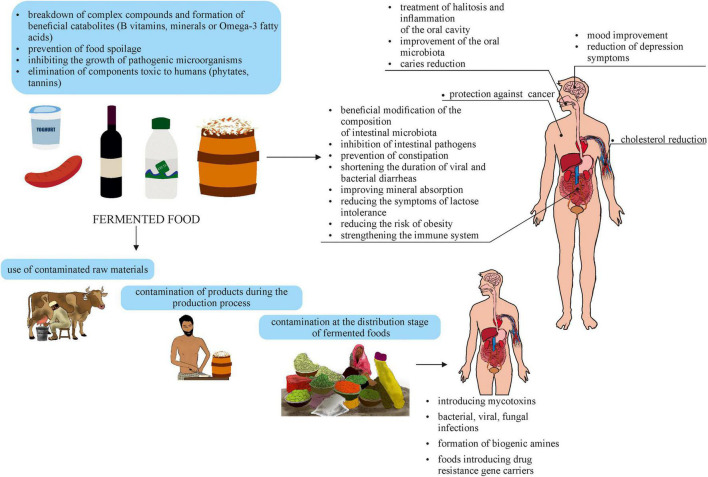
Effects of consuming fermented foods.

The health-promoting effect of fermented products is due to the presence of functional microorganisms in them. Microorganisms can occur naturally in various products (e.g., genera *Lactobacillus, Lacticaseibacillus, Levilactobacillus*) or, having GRAS (Generally Recognized As Safe) status, can be added to them (e.g., bacteria of the genus *Bifidobacterium*). The beneficial effect of microorganisms present in fermented products can be multidirectional (Figure 1). Since the consumption of functional foods can play a positive role in gut dysfunction, research is being conducted to determine their use in intestinal diseases. A study by Zhang et al. (2016), has shown that microorganisms present in fermented foods can transiently affect the gut microbiome. This allows for its modification and modulation of intestinal function, improving the health or reducing the risk of diseases associated with dysbiosis. Food can be a vehicle for probiotics, prebiotics, or synbiotics (Milani et al., 2019). The beneficial effects of probiotic strains include normalization of the gastrointestinal microbiota, antagonistic effects against pathogens, protection against pathogens’ colonization, short-chain fatty acid production, or metabolism of bile acid salts. Such properties make probiotics useful in intestinal diseases treatment (including *Clostridioides difficile* etiology), in the treatment and prevention of obesity, lactose intolerance, diabetes, osteoporosis, and cardiovascular diseases. An example of the positive effects of fermented foods on the intestinal al microbiota is alleviation of symptoms of irritable bowel syndrome resulting from the consumption of fermented probiotic milk containing *Bifidobacterium lactis* CNCM I-2494 (Marteau et al., 2013). Studies have also confirmed an improvement in gastrointestinal passage and a decrease in common complaints in the human population, such as bloating and flatulence. This may be related to changes in the expression of bacterial genes that encode enzymes involved, among others, in carbohydrate metabolism (Agrawal et al., 2009; McNulty et al., 2011). The importance of probiotics in enhancing non-specific and specific immunity (modulation of the host immune response) is also highlighted. Probiotic bacteria stimulate the mucosa-associated lymphoid tissue (MALT) immune system, formed, among others, by gut-associated lymphoid tissue (GALT) immune elements. Due to the production of chemokines, cytokines, growth factors, or immunoglobulins, MALT acts as a microbial fighter. Furthermore, probiotics influence the balance of the gut microbiome composition, reducing the risk of disease gut (Tokarz-Deptuła and Deptuła, 2017; Azad et al., 2018; Li et al., 2019; Uusitupa et al., 2020; Zhang et al., 2021). As microbes are able to produce neurochemicals, as well as respond to them, they can play a crucial role in the treatment of depressive and anxiety disorders (Romijn et al., 2017). Furthermore, the consumption of fermented products has a positive impact on the oral microbiota. The functional bacteria in the food reduce tooth decay, gum disease, and oral inflammation by lowering pH and producing antioxidants that inhibit plaque growth. They are also used in the treatment of halitosis, as they metabolize volatile sulfur compounds the source of unpleasant mouth odor (Gungor et al., 2015; Voidarou et al., 2020).

The microorganisms present in fermented products with high titers can interact with microorganisms that inhabit the digestive tract and colonize it temporarily or permanently (Davoren et al., 2019; Nemska et al., 2019; Roselli et al., 2021). Currently, due to the high degree of the variability of studies (heterogeneity of study design and methods used), and interindividual variability in the composition of the gastrointestinal microbiota, there is insufficient evidence for permanent colonization of the human gut by food microorganisms. However, the transient colonization shown in some cases indicates the need for permanent introduction of fermented products into the diet to maintain the positive effects of strains on the human body (Roselli et al., 2021).

The fermentation process is generally carried out to obtain a nutrient-enhanced product. However, in some cases, the overriding purpose is to prevent food spoilage. The metabolites produced by microorganisms (lactic acid, acetic acid, hydrogen peroxide, ethanol, compounds with antagonistic properties to other microorganisms) inhibit the growth of pathogenic microorganisms or those responsible for food spoilage. Fermented food is of great importance in low- and middle-income countries (subregions of Africa and Southeast Asia), which typically lack access to refrigeration facilities. In countries where the interplay of the dry season and the growing season results in a lack of availability of fresh food, preservation is a necessary solution to protect the population from starvation. The advantage of fermentation is also the ability to eliminate various types of toxic components present in raw materials, such as polyphenols (e.g., pachyrrhizine, rotenone, catechin derivatives), phytates, and tannins (Montagnac et al., 2009; Lautié et al., 2013). An example is the reduction of up to 95% of lectins and other toxic components in *tempe* produced from soybeans (Uzogara et al., 1990; Evans et al., 2013). It is possible to eat fermented products that would not be suitable for consumption without proper preparation (e.g., *cassava*, due to its cyanogen content) (Agbor-Egbe and Mbome, 2006). The increased digestibility of vegetable protein, by its partial breakdown, decreases the risk of food allergies and gastrointestinal disorders. An additional advantage of fermented products is their reduced mass, compared to the initial raw material, resulting from the processes (e.g., grating, soaking, squeezing) it undergoes before fermentation. This facilitates the transport of products which, especially in developing countries, is at a low level. In addition, the heat treatment time relative to the cooking time of the raw substrate is reduced (Uzogara et al., 1990; Nkhata et al., 2018).

Fermentation as a food processing technique can influence the level of mycotoxins in food. Mycotoxins pose a serious threat to human health due to their demonstrated carcinogenic, mutagenic, nephrogenic, hepato-, cytotoxic, neurotoxic, and teratogenic effects, and induction of immunosuppression. Toxins detected in foods produced by genera such as *Aspergillus, Penicillium*, and *Fusarium* include, among others, ochratoxin A, aflatoxins, zearalenone, and trichothecenes (Raiola et al., 2015; Ostry et al., 2017; Pei et al., 2021). Many strains of LAB producing antifungal metabolites (lactic acid, phenyllactic acid, hydroxyphenyllactic acid, indole, bioactive peptides) can reduce both fungal growth and mycotoxin synthesis. In addition to the inhibitory effect of bacterial organic compounds, antifungal activity may also be related to competition for the occupied niche and nutrients needed for growth. Modification of the external environment is also important here, as well as the binding of mycotoxins by components of the cell wall (polysaccharides, peptidoglycans) of bacteria. The species for which such properties have been demonstrated include strains of the genus *Lactobacillus* (e.g., *L. rossiae, L. fermentum L. sanfranciscensis*), as well as other bacterial genera such as *Bifidobacterium, Lactococcus, Pediococcus* (Valerio et al., 2009; Adedokun et al., 2016; Luz et al., 2017; Aarti et al., 2018; Guimarães et al., 2018; Khattab et al., 2018; Sivamaruthi et al., 2018; Sadiq et al., 2019).

Consumption of fermented foods also alleviate the severity of symptoms of COVID-19. This is due to the lactobacilli that are potent activators of nuclear factor (erythroid-derived 2)-like 2 (Nrf2), a major regulator of the cellular oxidative stress response (Bousquet et al., 2021). However, more this correlation merits further studies.

## Problems

External and internal factors affect the growth capacity of pathogenic microorganisms in fermented foods (Figure 1). The risk of obtaining a contaminated fermented product increases when low-quality ingredients are used for its production, initially containing a sufficiently high number of bacteria, fungi, or toxins produced by them. An example is the pork used to make *nem chua*, a traditional raw sausage eaten in Vietnam after a short spontaneous fermentation process. Research by Le et al. (2012) showed repeatedly exceeded the level of microbiological contamination in meat intended for *nem chua* production. The presence of *Escherichia coli* and *Staphylococcus aureus* detected in the raw material did not meet the requirements for hygiene and safety. In countries with high poverty, raw materials of better quality are used mainly for export, as they are the primary source of income. On the contrary, secondary crops are used in household or small-scale food production, resulting in products of inappropriate microbiological standards.

The water used in the dilution stage or in the fermentation itself should also be free from microbiological contamination. Unfortunately, limited access to water in some regions, especially in rural areas and the use of potentially contaminated water from streams or rivers for production, increases the risk *E. coli* and *Salmonella* spp. presence in food (Anyogu et al., 2021).

In developing countries, the lack of Good Manufacturing Practices (GMPs), has a major impact on the safety of traditional, home-made, or cottage-made food (Oguntoyinbo, 2014). Their sale under unsanitary conditions without the use of protective coverings, such as gloves, is also a public health risk. Moreover, in developing countries, due to poverty and low consumer awareness, fermented foods sold locally are usually packaged in non-sterile utensils, used jute bags, or paper (e.g., newspaper), as well as gourds, or leaves. The inability to buy adequate packaging to limit microbial spoilage, even with a properly executed production process, poses a significant additional risk of food contamination (Oguntoyinbo, 2014).

**FIGURE 2:**
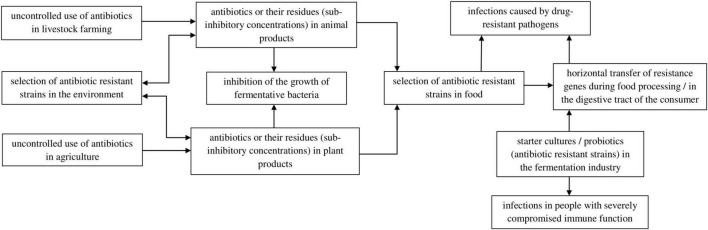
Factors responsible for strain resistance to antibiotics in food and its effect.

Despite the positive impact of fermented products on human health, there is a risk that their consumption can introduce into the body carriers of antibiotic and chemotherapeutic resistance genes, leading to the selection of multidrug resistant strains responsible for infections that are difficult to treat. This selection also occurs as a result of the overuse of antibiotics in agriculture and livestock farming. Bacterial resistance mechanisms can be generated by the presence of drugs or their residues in food products at concentrations below the minimum inhibitory concentration (MIC). Due to horizontal gene transfer (transformation, conjugation, or transduction), there is a risk of spreading drug resistance among gastrointestinal microorganisms or foodborne pathogens (Figure 2; Tóth et al., 2020, 2021; Miranda et al., 2021). Therefore, in the case of fermented food production, in addition to the prudent use of antibiotics by breeders and farmers, it is important to use strains that do not contain drug resistance genes in their genetic material or to monitor the content of these genes in starter cultures and manufactured products.

**FIGURE 3:**
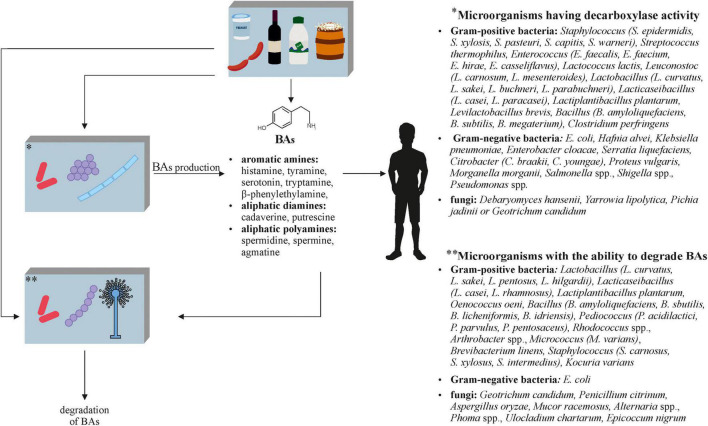
The role of microorganisms in the metabolism of biogenic amines (BAs) in fermented foods (Smith, 1980; Latorre-Moratalla et al., 2010; Lorenzo et al., 2010; Spano et al., 2010; Cueva et al., 2012; Alvarez and Moreno-Arribas, 2014; De Mey et al., 2014; Zaman et al., 2014; Ladero et al., 2015; Ordóñez et al., 2016; Sun et al., 2016; Pugin et al., 2017; Ekici and Omer, 2018; Li et al., 2018; Barbieri et al., 2019; Mah et al., 2019; Park et al., 2019; Ruiz-Capillas and Herrero, 2019).

The negative aspects of fermented foods also include biogenic amines (BAs) (Figure 3), which are formed during fermentation with both microorganisms naturally present in the raw material and with the participation of starter cultures. BAs are a product of amino acid decarboxylation, amination of aldehydes or ketones, or their transamination, and therefore their formation is influenced not only by the type of microorganism that performs these processes, but also by the composition of the raw material (e.g., free amino acid content), the fermentation time, and the conditions under which food processing takes place. Due to the higher accumulation of BAs in products that do not meet microbiological standards, it is believed that the amounts of these compounds may reflect the degree of spoilage (Doeun et al., 2017; Xiang et al., 2019). In order to reduce the BA content, it is necessary to use microorganisms or processes leading to the degradation of these potentially toxic compounds in food production. However, the main activities are to control the quality of the raw materials that undergo fermentation and its conditions, but they are not always carried out.

Adverse health effects associated with the consumption of fermented foods can include isolated illnesses, outbreaks, and even deaths. Among other things, due to the mycotoxins present in some products, long-term consequences are also possible in the form of diseases of the gastrointestinal tract, kidney stones, or cancer (Singh et al., 1986; Phukan et al., 2006; Gajamer and Tiwari, 2014; Keisam et al., 2019). The actual number of infections and outbreaks resulting from the consumption of fermented foods is probably underestimated. In developed countries, difficulties in detecting outbreaks that may originate from a variety of food products are associated with incomplete epidemiological data (mild cases of infection are not reported or documented) and the frequent lack of information exchange between diagnostic laboratories. This leads to delays in investigations and the inability to confirm hypotheses about a potential source of infection.

The composition of some fermented products may also cause some minor health disadvantages. An example of a product of this type can be kombucha made from tea and sugar. Its consumption can lead to excess sugar and calorie intake, which may also lead to bloating and gas.

## Dairy Products

Many food products, such as yogurts, kefirs, and cheese, are obtained from milk, usually cow’s milk, as a result of the fermentation process. Due to the diversity of the microbiome, various fermentation routes can be carried out, which leads to the production of thousands of final products (such as cheeses), differing in consistency, taste, and aroma (Voidarou et al., 2020).

Raw and fermented camel milk, which has high nutritional value, is consumed by the populations of Asian and African countries. It is considered a product with therapeutic properties because of the immunoglobulins, lysozyme, and lactoferrin content, which have antimicrobial activity. In some regions, it is used to treat diseases such as chronic hepatitis (Saltanat et al., 2009; Sharma and Singh, 2014). *Suusac*, consumed by the people of the arid and semi-arid areas of Kenya, is obtained from the spontaneous fermentation of fresh unpasteurized milk in cleaned smoke-treated gourds (Lore et al., 2005). A study by Maitha et al. (2019) on milk samples collected from different areas of northeastern Kenya found that *suusac* consumption also poses health risks to consumers. Researchers found *E. coli* in all samples analyzed, while *Shigella* spp. and *Klebsiella* spp. were present in 88.1 and 77.4% of the samples, respectively. Furthermore, bacteria of the species *S. aureus*, which are often responsible for animal udder infections, including camels, were detected in more than half of the samples. Another study reported on the detection of *Mycobacterium* strains other than *tuberculosis* (MOTT) (*Mycobacterium avium, M. intracellulare, M. kansasii, M. malmoense*) in *suusac* (8.2% of positive samples) (Mwangi et al., 2017). The authors of the above study also highlighted the problem related to the widespread distribution of *Brucella* spp. rods in dairy products in African countries. They showed that the risk of these microorganisms increases from 14 to 26% in *suusac* production. The ability of some *Brucella* species to survive at 4°C and at pH below 4.0 indicates the need for vaccination of livestock against brucellosis (Zúñiga Estrada et al., 2005; Dadar et al., 2019). Pasteurization of milk before *suusac* production or heat treatment before consumption of the finished product is also crucial to consumer health. Also, Iran by Yam et al. (2014) conducted a research aimed at determining the microbiological quality of fermented camel milk, *chal*out in (2014). *Chal* is obtained, like *suusac*, by spontaneous fermentation, carried out in leather bags or bottles. In samples collected in the province of Golestan, *Staphylococcus* spp. and rods from the Enterobacterales order were identified, while no bacteria of the genus *Salmonella* and *Shigella* were detected.

In addition to *mastitis*, poor hygiene of the milking personnel, the milking environment (milking is usually conducted in the open areas without prior washing and disinfection of the teats), and the fermentation tanks also contribute to milk contamination. The quality of the water used for washing is important, as it should be drinking water quality. Greater risk of infection poses a cheap product, kept in poor sales conditions. Fermented milk can be a particularly significant source of infection, as it is often consumed without heat treatment.

Among the microorganisms present in fermented milk are strains of *E. coli*. A study by Yakubu et al. (2018) showed the presence of the most pathogenic serotype *E. coli* O157: H7 in samples of this product from 100 points of sale in the area of Nigeria. The main source of *E. coli* strains, including those with a high pathogenic potential, is the gastrointestinal tract of cattle (animals do not show clinical signs of infection). These microorganisms, excreted in the feces, can contaminate milk, especially if premilking hygiene is not practiced (Leedom, 2006).

Many other studies have confirmed the contamination of products obtained by cow’s milk fermentation. An example is *roub*, a drink obtained by inoculating milk with starter culture from the previous day’s fermentation. It is drunk when diluted with water or added to make a soup to be eaten with pudding. In studies conducted on traditionally prepared *roub* samples collected in three regions of Sudan, a high level of contamination and the presence of *S. aureus* and coliform bacteria were demonstrated (Abdalla and Hussain, 2010). This confirms previously reported results (Abdalla and El Zubeir, 2006). Dehkordi et al. (2014) assessing the presence of shiga toxin-producing *E. coli* strains in traditionally produced dairy products (yogurt, *doogh*, and *kashk*) sold in supermarkets or retailers in Iran. The characteristics of the strains isolated from more than 8% of the products showed diversity in terms of serogroups, among which the serogroup reported the most frequently was O157 (26%) and O26 (12%). The relatively high frequency of strains responsible for various forms of infection, including bloody and non-bloody diarrhea or hemolytic uremic syndrome (HUS), may indicate failure to maintain the appropriate temperature or time parameters at the dairy production stage. It may also result from the use of contaminated water by the local population, who do not have access to water that meets microbiological requirements.

Nahidul-Islam et al. (2018) analyzed traditional hand-made fermented dairy products (*dahi, chanar-misti, paneer*, and *borhani*), produced in India but also popular in other Middle East to South East Asian countries. Their research with the use of pyrosequencing showed the presence in food, in addition to the dominant LAB (*Lactobacillus* spp. and *Streptococcus* spp.), differently numerically (depending on the product) bacteria, including *Acinetobacter* spp. and *Pseudomonas* spp., rods from Enterobacterales, as well as fungi of the genus *Aspergillus*. The probable sources of these microorganisms are skin, bovine intestine, and *mastitis*.

*Ras* and *karish* cheese showed significant contamination with *E. coli* strains (8–21.7% and 74.5%, respectively) (Ombarak et al., 2016; Hegab et al., 2020). These cheeses are consumed in Egypt, a country where fresh milk and products thereof are important components of the daily diet. The cheeses are usually obtained in artisanal rural areas from raw cow’s milk or a mixture of cow’s and buffalo milk. Fermentation and ripening, which take 3–8 months, usually involve only microorganisms that are native to the milk microbiota. *E. coli* strains isolated from cheese possessed different pathogenic potential. Some strains, were responsible for *gastroenteritis*. The most common source of these microorganisms in raw milk and dairy products derived from it is feces that contaminates the milk at the milking stage. Hegab et al. (2020) also noted *S. aureus* strains in 26% of *ras* cheese samples, 15% of which were enterotoxigenic (demonstrated presence of *seb* and *sed* genes). Abdel-Hameid Ahmed et al. (2019) found an even higher percentage (50%) of enterotoxin producing *S. aureus* strains isolated from this type of cheese. The above results may indicate poor hygiene of people involved in the production of cheese.

In developing countries, pathogens that have been largely eliminated in other parts of the world may also be present in fermented dairy products. Examples include the previously mentioned *Brucella* spp., or MOTT, found in milk. Based on a study by Michel et al. (2015), *Mycobacterium bovis*, an etiological agent of bovine tuberculosis, can survive in souring cow’s milk. This species is isolated in Africa from unpasteurized milk, but data on the incidence of the disease in humans are insufficient to conclude how high the risk of consuming contaminated products with *M. bovis* is. Inappropriate veterinary control of livestock in some countries is certainly a significant cause of approximately 70,000 cases of zoonotic tuberculosis reported annually in Africa (Olea-Popelka et al., 2017; Owusu-Kwarteng et al., 2020).

Despite the presence of many viruses in food, infections of this etiology caused by the consumption of contaminated fermented products are recorded less frequently than bacterial infections. This is due to the fact that viruses present in food are primarily bacteriophages and yeast-infecting viruses (Pringsulaka et al., 2011; Kleppen et al., 2012). The low number of these microorganisms in food products and the greater difficulty of detecting them may also be a reason. Foodborne viruses include *hepatitis* A and E virus, noroviruses, and rotaviruses (Maske et al., 2021). In 2016, two cases of tick-borne encephalitis were reported in Germany after consumption of unpasteurized goat cheese (Brockmann et al., 2018). Infections caused by tick-borne *encephalitis* virus transmitted by this type of cheese, although rarely, have also been found in other countries of Central and Eastern Europe (Croatia, Czech Republic, Austria) (Holzmann et al., 2009; Kríz et al., 2009; Markovinović et al., 2016). The work of Rehfeld et al. (2017) suggests that in Brazil, where cow’s milk contamination with the Vaccinia virus (VACV) is noted, consumption of artisanal cheese from unpasteurized milk may also lead to illnesses of this etiology. The route of transmission of SARS-CoV-2 through food consumption has not been confirmed, but is considered unlikely (Center for Disease Control and Infection, 2020).

## Products of Plant Origin

Various substrates of plant origin, such as cereals, oil seeds, nuts, roots, tubers, and plant juice, are fermented. Some of them are an important and inexpensive source of protein, which provides energy for the body. Additionally, the breakdown of proteins into amino acids during the fermentation process increases the digestibility of the product.

Sorghum is the second most commonly grown cereal in sub-Saharan Africa, thanks to its tolerance to drought, its ability to grow under harsh conditions, and its nutritional value (high starch content, among other things). Due to its benefits, it is an ingredient in the main meals consumed by the inhabitants of Africa. In addition, it is an important component of the diet of people with gluten intolerance. However, a fermentation process is required to convert the plant into an edible form (Odunmbaku et al., 2017; Adebo, 2020). The most common fermentation process in sorghum is lactic fermentation, in which primarily LAB participate, although certain fermented products involves also fungi. One of the traditionally produced sorghum-based products (or millet) is *obushera*. Due to its widespread use (in weaning, as a thirst-quenching drink and as a source of energy), its production is a source of income for households, but it is also being commercialized. *However, obushera* sold on the market does not always meet microbiological requirements. In the study by Byakika et al. (2019), despite the absence of *Salmonella* spp. rods in the samples tested, most did not meet the standards for coliforms and *Staphylococcus* spp. Microbiological contamination could result from the use of poor quality raw materials in production, but also by the lack of pasteurization processes.

Adedeji et al. (2017) and Ademola et al. (2018) conducted studies on *iru* and *ogiri*, traditional food condiments used in Nigeria and some parts of West Africa. They are popular due to the aromas and flavors, resulting from the primary and secondary metabolites of microorganisms produced during fermentation, and their high protein content, which is of a particular importance in the poor regions of the country. The first of these condiments is obtained from carob seeds [African locust bean (*Parkia biglobosa*) seeds], while the raw materials for *ogiri* may be melon seeds or castor bean seeds. The production of condiments is preceded by spontaneous fermentation of seeds, usually carried out at the household level. Previously conducted analysis of these products, using classical methods of bacterial identification, based on their biochemical characteristics, showed the presence of potentially pathogenic bacteria, such as *Bacillus cereus, Bacillus subtilis, S. aureus, E. coli, Proteus* spp., *Pseudomonas* spp. (Falegan, 2011; Ajayi, 2014). Adedeji et al. (2017) and Ademola et al. (2018) extended the research to include genotyping methods, confirming the participation of various microorganisms in condiments, representing both actual fermenters and undesirable species introduced at different stages of production. Ademola et al. (2018), analyzing the dynamics of the bacterial population during the production of *iru* and *ogiri*, showed that species belonging to the genera *Bacillus (B. encimensis* and *B. safensis*), *Enterococcus (E. dispar)*, and *Lysinibacillus* are present at almost every stage of spice processing. Therefore, they can be potential starters in the fermentation process. Other microorganisms, including potential pathogens, detected only at certain stages of processing are evidence of poor hygiene practices leading to contamination of foods that play an important role in the diets of rural populations in poor countries.

*Douchi* is a Chinese condiment obtained from black beans. In its production, strains of *Aspergillus* spp., *Mucor* spp. or *B. subtilis* are used, carrying out the first fermentation process, while the second process is an anaerobic spontaneous fermentation, with the participation of bacteria and fungi (Zhang and Liu, 2000). *Douchi* was the cause of an outbreak of food poisoning that occurred in Kunming, China (Zhou et al., 2014). The symptoms of food poisoning that occurred in 139 people who consumed a dish containing *douchi* were caused by cereulide or Nhe enterotoxin produced by strains of *B. cereus*. However, this was not the first case of *douchi*-related food poisoning reported in China. A two other previously reported outbreaks, *B. cereus* strains were also the etiological agent (Shen et al., 2005; Liu et al., 2006).

Species such as *B. cereus, Clostridium botulinum*, *Proteus mirabilis*, and *E. coli* have been detected in fermented soy products. Keisam et al. (2019) carried out analysis of such products sold in the northeastern region of India was using the next generation sequencing technique (MiSeq) combined with qPCR and immunoassays. The first two species mentioned were found in all samples taken for testing in the amount of > 10^7^ cells/g. In addition, diarrheal or emetic toxins (hemolysin BL (HBL) and non-hemolytic enterotoxin (NHE) were detected in all isolated strains of *B. cereus*, while cereulide (an emetic toxin) was detected in less than half. In turn, the strains of *P. mirabilis* produced hemolysins, urease, and also showed multidrug resistance. The common presence of these intestinal rods in fermented foods could explain the high percentage of cases of *urolithiasis* in India.

The contamination of fermented soybean products with *B. cereus* strains is also a significant problem in Korea, where these products are common in the daily diet. *Doenjang, kochujang, meju*, or *cho-kochujang* are obtained by natural fermentation, either at home or with factory-made starter cultures. Its pro-health properties, such as its anticancer effect, has led to an increase in interest and frequency of its consumption not only among Koreans, but also among people around the world (Jung et al., 2006; Lee N. et al., 2017). However, numerous studies have confirmed the presence of strains of *B. cereus* in such products (Kim et al., 2015; Yim et al., 2015; Park et al., 2016; Lee N. et al., 2017). Although the detection rate of this bacilli species, as well as the level of product contamination, were varied (not always exceeding the acceptable standards of the Korean Food and Drug Administration), the NHE and/or HBL toxins detected in the strains indicate the need to monitor the level of *B. cereus* contamination in soybean products.

In South Korea, two outbreaks of *gastroenteritis* caused by enterotoxigenic *E. coli* (ETEC) O6 strains were reported in 2013–2014 (Shin et al., 2016). The first included 167 children attending a middle school in the Jeollanam-do province, and the other involved 1,022 cases in 10 schools in the Incheon Provence. *Kimchi* was suspected to be a carrier of pathogenic strains. This traditional Korean dish is prepared by fermenting various types of vegetables, the most popular of which is cabbage. The product is consumed about 1 week to several months after its preparation and contains beneficial bacteria that carry out the fermentation process, mainly from the genera *Leuconostoc, Lactobacillus*, and *Weissella*. Epidemiological and laboratory investigations reported ETEC O6 strain in *kimchi* prepared from cabbage in the school canteen and young radish prepared by a food company. A potential carrier of the bacteria could be poor quality water used for food preparation. Both, too short fermentation process and established environmental conditions may allow pathogens to grow of intestinal pathogen. Furthermore, excessive pH does not inhibit the secretion of the heat-labile (LT) toxin responsible for diarrhea by ETEC strains, as shown in a study by Gonzales et al. (2013). *Kimchi* prepared from cabbage or radish for the canteens of 7 schools in Incheon, Korea, was also likely the cause of a previous large outbreak of 1,642 cases of *enteritis* (Cho et al., 2014). Retrospective cohort studies carried out allowed the isolation of ETEC O169 strains indistinguishable in pulsed field gel electrophoresis (PFGE) from 230 students and kimchi produced by one food company. However, the presence of these pathogens was not found in raw vegetables or other food products. The reported outbreaks are evidence that not only homemade fermented foods, but also foods produced by food companies, may pose a risk of infection for the consumer. Hence, there is a need for continuous monitoring of food safety and the need to define precise criteria for the production process in food establishments. This is especially important in the case of a wide distribution of products due to the risk of causing a large outbreak in a relatively short period of time.

The concentration of mycotoxins depends on their initial content in the raw material. Therefore, in low-income countries, due to the frequent use of low-quality cereals, mycotoxins are found in products obtained from fermented crops. The presence of fungi producing toxic metabolites is influenced by the timely failure to clean crops from the soil, as the well as long-term and improper (at high humidity) storage of grains, especially crops without hulls. Also, crops damage during harvesting or storage increases the risk of mycotoxins in raw material. Favorable environmental conditions, such as temperature or water activity, are important factors that contribute to mycotoxins production (Milani and Maleki, 2014; Viaro et al., 2017). The risk of consuming fermented products prepared from raw material contaminated with mycotoxins is high, even with previous heat treatment, due to the stability of these toxins at processing and cooking temperatures.

People’s awareness, mainly in developing countries, of the food contamination risk with fungi and mycotoxins is low (Siegrist and Cvetkovich, 2001; Ezekiel et al., 2013; Matumba et al., 2016; Adekoya et al., 2017). Therefore, few activities, such as sorting moldy seeds or proper drying, are undertaken to reduce the contamination of raw materials used in food production, as well as the contamination that can occur during its preparation or storage. Ignorance or neglect by sellers, in the turn, results in mixing products that meet microbiological requirements with moldy food. Mycotoxins are often found in various types of products typically consumed in African countries, produced based on spontaneous fermentation or small-scale rural processing. In a study by Adekoya et al. (2017), more than 80% of the samples analyzed of foods produced in Nigeria and obtained by fermentation of raw materials such as melon seed (*Citrullus colocynthis*), oil bean seed (*Pentaclethra macrophylla*), maize or sorghum contained single toxins or combinations thereof. Some of them (fumonisin, aflatoxin, ochratoxin A, and zearalenone) exceeded the limit specified by the European Commission.

In China, fermented pastes, made from soybeans, broad beans, flour, and chili, are of great interest. Contamination with fungi and the formation of mycotoxins often occur during the cultivation stage if the species of fungi are compatible with the crop. However, studies have shown that mycotoxin production in this type of food can also occur during a long fermentation process (Shukla et al., 2014). Aflatoxins (present in soybean pods and seeds) and ochratoxins (present in wheat and soybeans) are frequently detected in fermented pastes (Zhao et al., 2020). The presence of aflatoxins was also found in soybean sauces obtained with the participation of *Aspergillus orizae* (Vu and Nguyen, 2016).

In Korea in 2013, *kimchi* produced by a food company was the source of acute *gastroenteritis outbreaks* caused by the GI.4 human norovirus (HNoV) genotype (Park et al., 2015). The analysis showed that the *kimchi* was probably contaminated with groundwater used by the company during the production stage. Spreading the product before the completion of the fermentation process may not have lowered the pH sufficiently, with the result that HNoV, which is resistant to pH > 5, was able to survive in *kimchi*. Research by Lee H. M. et al. (2017) also showed that despite the reduction of the HNoV titer in experimentally contaminated cabbage *kimchi*, the fermentation conditions (acidity, salinity, organic acid content) may be an insufficient factor to eliminate this virus.

## Meat Products

Due to the high likelihood of pathogens in raw meat, the risk of infection after consumption of fermented meats not heat treated prior to consumption is high. The addition of nitrite to meat helps reduce the growth of microorganisms such as *C. botulinum, S. aureus, L. monocytogenes*, and *Salmonella* spp. (Hospital et al., 2016; Majou and Christieans, 2018). However, since these compounds are an important risk factor for colorectal cancer, there is a trend toward eliminating them from the meat industry and introducing alternative preservation methods. This, however, may lead to a lower microbiological safety of the product (Christieans et al., 2018; Gonzalez-Fandos et al., 2021).

One pathogen most frequently reported responsible for foodborne disease outbreaks associated with meat products is *Salmonella* spp. (Patarata et al., 2020). In Italy, an outbreak involving 79 cases occurred in 2009–2010, caused by the Goldcoast *Salmonella enterica* serotype (Scavia et al., 2013). It is believed that its source could have been salami, dry, fermented sausages. Nonetheless, a delayed investigation, carried out after the peak of the outbreak, did not allow to confirm this suspicion. Stool samples collected from the patients for diagnostic testing were too small. It was also difficult to take samples of suspect food products and examine the trace-back activity of food, especially in the case of salami, produced from the meat of various species of animals. Furthermore, various typing methods were used in different laboratories. Despite low pH and water activity, as well as high salinity, *Salmonella* may remain in salami due to the too short fermentation period. The salami production process reduces significantly *Salmonella* spp. levels but the scale of reduction with high primary meat contamination may be insufficient. Cases of increased infections (60 cases of diarrhea, abdominal cramps, or fever) of *Salmonella* Goldcoast etiology were reported at a similar time in Hungary, where contaminated pork was the likely source (Horváth et al., 2013). In turn, raw pork and fermented raw pork sausage, Zwiebelmettwurst, were the likely vehicle for the transmission of *Salmonella enterica* serovar Bovismorbificans during the German outbreak (Gilsdorf et al., 2005). At that time, 525 cases of *gastroenteritis* were reported, with one death.

Dry fermented sausages are highly diversified, influenced, among others, by the degree of grinding meat and fat, its acidity, or the presence of mold on the surface (Meloni, 2015). Although listeriosis outbreaks associated with the consumption of these types of meat are rarely reported (Meloni, 2019), *L. monocytogenes* is relatively frequently detected in final products, demonstrating the ability of these bacteria to overcome barriers at the production stage. The source of *L. monocytogenes* in fermented sausages can be raw meat, the slaughterhouse environment, or people in contact with the raw material unprocessed or after processing. Resistance to disinfectants and the ability to form biofilms on various types of surfaces increase the risk of contamination of ready-to-eat (RTE) products (Martin et al., 2011; Meloni et al., 2014; Meloni, 2015).

Díez and Patarata (2013) studied the pathogen’s survival during the production of *chouriço*, a dry fermented sausage. This product, which is produced both on a farm scale (using natural fermentation) and on an industrial scale (fermentation carried out under controlled conditions, using starter cultures), differs not only in the spices added, but also in the smoking, drying, and maturation times, which last between 1 and 4 weeks. An important role in meat preservation plays the amount of salt, which reduction for health reasons in recent years simultaneously decreases the microbiological safety of the product. The provocation tests carried out by Díez and Patarata (2013) showed that although the levels of *L. monocytogenes, S. aureus*, and *Salmonella* spp. were reduced in the early stages of drying, all pathogens were undetectable in *chouriço* after a longer period (30 days) of this process. In addition to salt concentration, a sufficiently high glucose content, the addition of antimicrobial compounds, or a low pH affect the survival of microorganisms (Piras et al., 2019). The conditions under which fermentation occurs are also important. The fermentation temperature and postproduction processing, including storage temperature and time, as well as product freezing and thawing processes, are crucial in reducing the content of toxigenic strains of *E. coli* (Heir et al., 2013; McLeod et al., 2016; Shane et al., 2018). Therefore, to ensure the microbiological safety of fermented meat, while maintaining its taste, it is necessary to optimize the ingredients added to it and the process parameters.

Lee H. S. et al. (2017) have suggested that feeding animals with feed contaminated with mycotoxins can contaminate meat products. The research was carried out in Vietnam, where the tropical climate (high temperature and humidity) is particularly conducive to fungal growth in a variety of agricultural products, including pig feed products. Evaluation of exposure of pigs to aflatoxins has shown their presence in the urine of animals, suggesting a risk of toxins’ presence in pork meat. However, the authors did not conduct any research in this regard.

Fermented meat products can also be a source of *hepatitis* E virus (HEV) infection, which is transmitted through meat from infected pigs or wild boar. In fact, a study by Wolff et al. (2020b) showed that despite the inhibitory effect of an acidic environment on many microorganisms, HEV shows a minimal decrease in infectivity at pH 2. Other studies by Wolff et al. (2020a) indicate that high salt concentrations that are usually applied for fermented raw sausages are also not sufficient to limit HEV survival in food products. Furthermore, an assessment of the prevalence of HEV genotype 3 in RTE products containing raw meat and originating from the Swiss retail market showed the presence of genetic material of these viruses in approximately 6% of samples (Moor et al., 2018). The RNA of HEV genotype 3 was also confirmed in a study of raw sausage samples collected from retail stores in the Netherlands (14.6% of samples) (Boxman et al., 2020). The greatest risk of infection with HEV etiology occurs when consumed raw pork products contain the liver of a contaminated animal (Pavio et al., 2014). This exemplified the hepatitis E outbreak reported in France associated with the consumption of figatelli, raw pig liver sausages (Renou et al., 2014). Since there is a high risk of pork meat contamination with the virus, it would be advisable to consider the HEV test, and the obligation to report cases of hepatitis E, which is currently not mandatory in many countries. In some European countries, raw sausages do not contain a pork liver (Vignolo et al., 2010). Another option is to inform people about the risk of consuming such foods without heat treatment.

## Fish Products

Fermented fish products, due to their protein content, are an important part of the diet in some countries such as Thailand, the Philippines, Cambodia and Indonesia. Salt and sometimes sun-drying are used for preservation, while microorganisms play a primarily role in developing the fish’s characteristic flavor and aroma. In Norway, *rakfisk*, a traditionally produced fermented fish product, is very popular. Its raw material is freshwater salmonid fish, which are stored in brine at 3–8°C for 3–12 months, during which the fermentation process takes place. The finished product does not require heat treatment before consumption and therefore microorganisms that exhibit tolerance to increased salt concentrations can pose a risk to the consumer (Skåra et al., 2015; Bjerke et al., 2019). An example of such microorganisms is *L. monocytogenes*, which adapts to environments with high salinity and low temperatures in which it can multiply. A study by Axelsson et al. (2020) showed that temperature and salt may not sufficiently reduce the growth of *L. monocytogenes* in *rakfisk*. Therefore, additional strategies in the production of fermented fish are necessary, such as the use of the P100 bacteriophage, which reduces the pathogen’s number and has GRAS status (generally recognized as safe) (Allende et al., 2016).

Raw oysters are one of the foods that transmit noroviruses and are responsible for several foodborne outbreaks of *gastroenteritis* (Le Guyader et al., 2006). The virus accumulates in the shellfish bodies during water filtration and is able to survive for a long time in oyster tissues. In South Korea and other Asian countries, raw oysters can be fermented before consumption. The process is carried out at room temperature for about 2 weeks in the presence of 5–10% salt. The HNoV outbreak, which affected 8 students at a high school in Gyeonggi Province (Cho et al., 2016), indicates that although the viral load decreases significantly during oyster fermentation, this reduction may not be sufficient to eliminate the risk of *gastroenteritis*, as confirmed by research by Seo et al. (2014).

Hepatitis A outbreaks that occurred in South Korea in 2019 have contributed to the research on the causal source of this disease. Jeong et al. (2021) showed that hepatitis A virus (HAV) strains were present in yogaejot, a traditional fermented food from that country that contains raw clams. Phylogenetic analysis confirmed that they are closely related to the strains prevalent in East Asia. The results obtained indicate the need for proper hygiene practices in the production stage. Furthermore, since an important source of HAV infection is fecally contaminated coastal waters where bivalve mollusks are harvested, it is necessary to improve regulations protecting against this activity.

## Alcoholic Beverages

Alcoholic beverages containing more than 0.5% v/v of alcohol are made from raw materials such as cereals, vegetables and fruits, palm juice or honey (Voidarou et al., 2020).

Bacteria can develop tolerance to the acid and alcohol contained in various fermented products, as exemplified by the results obtained by Gómez-Aldapa et al. (2012). These researchers evaluated the behavior of *E. coli* O157: H7 during the fermentation from nectar of maguey agave plants used to make *pulque*, a traditional Mexican alcoholic beverage. The presence of *E. coli* O157: H7 strains in the *pulque*, although not proven, is highly probable. Although some companies industrialized the production of this beverage, the most commonly consumed is *pulque* made by artisans, also involved in cattle and sheep farming. These animals constitute an important reservoir of pathogenic *E. coli* strains and, often grazed on agave plantations, can contaminate the plant (Moxley, 2004; Varela-Hernández et al., 2007). Also importantly, *E. coli* O157: H7 strains can develop an adaptive response to acidic environmental conditions and survive the fermentation process (Bachrouri et al., 2006). Moreover, Gómez-Aldapa et al. (2012) showed that these bacteria can exhibit tolerance to alcohol at low pH, and thus can be present in finished *pulque* posing a high potential risk to the consumers’ health.

*Pombe* is an alcoholic beverage obtained from a variety of raw materials such as corn, millet, bananas, and pineapples (Kubo, 2016). In 2015, there was an outbreak in a village in Mazambique associated with the consumption of *pombe*, prepared from maize flour (Falconer et al., 2017; Gudo et al., 2018). Of the more than 230 people who developed symptoms of food poisoning and respiratory problems, 75 people died. The analysis revealed the presence of potentially lethal levels of bongkrekic acid, a potent toxin produced by *Burkholderia gladioli* pv. cocovenenans strains, in the collected beverage samples. This highly unsaturated tricarboxylic fatty acid can block the mitochondrial adenine nucleotide translocator (ANT) and prevent respiratory chain phosphorylation. The severity of symptoms caused by bongkrekic acid depends primarily on the amount of toxin-containing product consumed (Peng et al., 2021). Although *B. cocovenenans* shows sensitivity to high temperatures, the toxin itself is thermostable contributing to high mortality rate. Therefore, cooking foods containing bacteria does not protect against the effects of bongkrekic acid. Furthermore, Falconer et al. (2017) concluded from their research that the presence of *Rhizopus oryzae* in the raw material could enhance the toxin synthesis. Mass illnesses associated with the consumption of fermented foods containing bongkrekic acid had been reported many years earlier, in Indonesia (following the consumption of coconut-based tempeh) (van Veen, 1967) and in China (following the consumption of homemade fermented corn flour products) (Meng et al., 1988). However, many more similar outbreaks could not have been detected due to the smaller scale or the lack of research. Also in China, homemade sour soup prepared from fermented corn flour caused the death of all nine people consuming it (Yuan et al., 2020). Inadequate storage and processing conditions of raw materials, especially those rich in oleic acid, which create a good environment for the production of toxins by these bacteria may enable growth of *B. cocovenenans*.

A study by Scussel et al. (2013) demonstrated the presence of various types of toxins in wines randomly collected from a retail market in the Netherlands. Ochratoxin A and penicillinic acid produced by *Aspergillus* spp. and *Penicillium* spp. as well as alternariol and alternariol methyl produced by the genus *Alternaria* were detected in the samples tested by liquid chromatography based on tandem mass spectrometry. Primarily the region in which the grapes are grown and the associated climatic conditions affect the presence of mycotoxins in wine. The type of wine is also important because, unlike white wine, fermentation in red wines begins with the peel, which may contain mycotoxins. Therefore, the level of toxins detected by the researchers varied and depended on the type of sample tested and the country from which the wine originated. Hence, even low contamination of wine, with regular or frequent consumption, characteristic for some countries (e.g., France, Italy), due to the carcinogenic properties of mycotoxins, can pose a significant threat to human health. Also cork can be a source of toxins in wine. Treatment of corks with fungicides can reduce the risk of toxigenic fungi, but contamination can also occur after processing. A consequence of this is the transfer of mycotoxins from the cork to the wine (Centeno and Calvo, 2002).

Drinking *tari*, the fermented sap of the date palm, may contribute to the transmission of Nipah virus. Research in Bangladesh (Islam et al., 2016) found that this product could have been responsible for three outbreaks that occurred between 2011 and 2014 in the country. As sap collection usually consists of hanging clay vessels on the palm, into which sap drips, it can easily become contaminated with excrements and bat secretions, which are the source of the virus. Despite the sensitivity of the Nipah virus to alcohol solutions, its content (5–8%) in the sap after fermentation is too low to eliminate this microorganism from the beverage. It is important to remember that the lack of strategies to prevent sap contamination during harvest by bat droppings and secretions can also lead to the development of other diseases caused by viruses for which these animals are an important reservoir. The use of protective bamboo covers as a barrier for animals can be an effective solution.

## Conclusion

The benefits of consuming fermented foods may be particularly important to people in developing countries where there is no access to probiotic. Such foods can be a source of good microbes, help reduce diarrhea, and stimulate the immune system to fight other microbes. However, despite many advantages that result from the fermentation processes with the participation of various microorganisms, especially functional ones, the lack of good production practices creates the risk of microbiological contamination of food products. This phenomenon is particularly visible in developing countries, where food processing is highly dispersed and individual, or there is no system and institutions supervising the processes of food production, including fermented ones. In this case, the risk of consuming fermented foods should be considered, especially if they are contaminated with pathogens, including viruses. The purchase and consumption of such foods by tourists poses a real risk of spreading infection around the world. Therefore, it is advisable to inform food handlers of the risks associated with the consumption of food contaminated with fungi, bacteria, the toxins they produce, and viruses. This will make individual producers want to pay more attention to the sanitary safety of the food they produce. Concerns about the quality of the raw material should also be recommended. A contaminated raw material will not produce a safe product, especially a fermented product that is made without heat treatment. Hence, it is necessary to ensure appropriate harvesting dates, pay attention to the weather during harvesting, reject batches of raw material, which visual quality deviates from the expected (serious mechanical damage, mold, discoloration, etc.), and avoid obtaining milk from animals manifesting symptoms of disease. Equally important is the concern for personal hygiene of those who work in harvesting/collecting, processing, packaging, and distributing food. For this purpose, it is necessary to increase access to clean water sources or to enable the use of portable sources. It is also crucial to ensure optimal storage conditions, especially the cleanliness of storage rooms, and to optimize its temperature. These relatively simple treatments will significantly reduce the spread of foodborne pathogens. Also, in developed countries, it is possible to improve certain procedures by strictly adhering to the HACCP system, good manufacturing and hygiene practices, and the appropriate design of food processing plants. It is also worth trying to introduce various types of innovative solutions, mainly in food packaging, but not only. Reducing the contamination of food products with mycotoxins is possible by using adsorption materials in animal husbandry or toxin-degrading microbial catalysts. Following these rules will allow producing microbiologically safe food and enjoying the benefits associated with the consumption of fermented products.

## Author Contributions

KS, AB, and KG-B: conceptualization and supervision. AB, EW-Z, and NW-K: writing—original draft preparation. KS, KG-B, MA, EW-Z, and EG-K: writing—review and editing. KS and AB: visualization. EG-K: funding acquisition. All authors have read and agreed to the published version of the manuscript.

## Conflict of Interest

The authors declare that the research was conducted in the absence of any commercial or financial relationships that could be construed as a potential conflict of interest.

## Publisher’s Note

All claims expressed in this article are solely those of the authors and do not necessarily represent those of their affiliated organizations, or those of the publisher, the editors and the reviewers. Any product that may be evaluated in this article, or claim that may be made by its manufacturer, is not guaranteed or endorsed by the publisher.
